# BDNF trafficking and signaling impairment during early neurodegeneration is prevented by moderate physical activity

**DOI:** 10.1016/j.ibror.2016.08.001

**Published:** 2016-08-30

**Authors:** Michael F. Almeida, Rodrigo S. Chaves, Carolliny M. Silva, Juliana C.S. Chaves, Karla P. Melo, Merari F.R. Ferrari

**Affiliations:** Department of Genetics and Evolutionary Biology, Institute for Biosciences, University of Sao Paulo, Sao Paulo, SP, Brazil

**Keywords:** TrkB receptor, Treadmill running, Hyperphosphorylated Tau, Early neurodegeneration, Aging, Hippocampus

## Abstract

Physical exercise can attenuate the effects of aging on the central nervous system by increasing the expression of neurotrophins such as brain-derived neurotrophic factor (BDNF), which promotes dendritic branching and enhances synaptic machinery, through interaction with its receptor TrkB. TrkB receptors are synthesized in the cell body and are transported to the axonal terminals and anchored to plasma membrane, through SLP1, CRMP2 and Rab27B, associated with KIF1B. Retrograde trafficking is made by EDH-4 together with dynactin and dynein molecular motors. In the present study it was found that early neurodegeneration is accompanied by decrease in BDNF signaling, in the absence of hyperphosphorylated tau aggregation, in hippocampus of 11 months old Lewis rats exposed to rotenone. It was also demonstrated that moderate physical activity (treadmill running, during 6 weeks, concomitant to rotenone exposure) prevents the impairment of BDNF system in aged rats, which may contribute to delay neurodegeneration. In conclusion, decrease in BDNF and TrkB vesicles occurs before large aggregate-like p-Tau are formed and physical activity applied during early neurodegeneration may be of relevance to prevent BDNF system decay.

## Highlights

• There is decrease in BDNF signaling in absence of protein inclusions in hippocampus of aged rats.
• Trafficking of BDNF/TrkB decreases in hippocampal cells in the absence of pTau.
• Moderate physical activity prevents the impairment of BDNF system in aged rats.

## 1 Introduction

Alzheimer's disease (AD) is the most common cause of dementia, characterized by progressive neurodegeneration. AD is characterized by the presence of amyloid-beta (Aβ) deposits in the form of senile plaques and the intraneuronal aggregation of hyperphosphorylated tau, a microtubule-associated protein, in hippocampus (Schindowski et al., 2008, Simic et al., 2016). These aggregates seem to be associated with a variety of cellular insults like synaptic failure, dysregulation of intracellular signaling cascades, disruption of axonal transport, oxidative stress and mitochondrial dysfunction (Hashimoto et al., 2003). However, it remains uncharacterized whether protein aggregation is the direct cause of neuronal death or there are cellular events that occur in the absence of protein aggregated and collaborate for cell damage.

It is well accepted that oligomers, which are the early stages of protein aggregation, might be more toxic than protein inclusions, since they may disrupt axonal transport as well as spread tau oligomeric seeds (Chung et al., 2001, Hoover et al., 2010, Lasagna-Reeves et al., 2010, Flach et al., 2012, Xie and Chung, 2012, Riemer and Kins, 2013).

Maintenance of neuronal function, neuroplasticity, morphogenesis and survival are ensured by axonal transport of vesicles, mitochondria, neurofilaments and other cell components (Decker et al., 2010, Hirokawa et al., 2010). Thus dysregulation of trafficking has significant physiological consequences in neurons, such as synaptic dysfunction. Moreover, early stages of AD are characterized by disruption in axonal transport, corroborating with the disease pathophysiology (Muresan and Muresan, 2009, Goldstein, 2012, Millecamps and Julien, 2013).

Several previous studies demonstrated decreased spine growth, synaptic transmission, loss of memory and learning difficulties by disruption on transport of brain-derived neurotrophic factor (BDNF) and its receptors (Kennedy et al., 2010, Lu et al., 2013, Yoshii et al., 2013).

BDNF is the major brain neurotrophin. It is involved in neuronal differentiation, maturation and survival; it modulates synaptic transmission and neuronal plasticity (Sebastiao et al., 2011). Intracellular signaling of BDNF involves activation of tropomyosin-related kinase B (TrkB) receptor, which may be associated to a tyrosine kinase intracellular domain (TrkB-full length; TrkB-fl) or independent of kinase activity (TrkB-truncated; TrkB-tc), and activation of PLCγ, PI3K/Akt, and ERK/MAPK pathways (Friedman, 2000, Patapoutian and Reichardt, 2001, Numakawa et al., 2010, Luine and Frankfurt, 2013, Kellner et al., 2014).

BDNF and TrkB-fl receptors levels decrease during aging (Webster et al., 2006, Diogenes et al., 2007, Lee et al., 2009), in AD their levels are further reduced (Ginsberg et al., 2006, Kao et al., 2012). Therefore, impaired transport and low expression of BDNF might contribute to synaptic dysfunction in AD (Khatri and Man, 2013, Scharfman and Chao, 2013). However, it remains poorly understood whether impaired axonal transport is cause or consequence of reduced BDNF signaling, and whether this is affected during the course of protein aggregation.

In neurons, anterograde trafficking of TrkB receptors is done by the interaction among Slp1, Rab27B and CRMP-2 proteins that direct link TrkB to the molecular motor Kinesin-1 (Arimura et al., 2009, Hirokawa et al., 2010). For retrograde trafficking, Pincher (EDH-4) and Rab5 are proteins that interact with the dynein/dynactin complex (Valdez et al., 2005, Zweifel et al., 2005, Hirokawa et al., 2010, Philippidou et al., 2011).

Physical exercise is postulated to promote neurogenesis through increase in BDNF levels, as well as increasing the levels of some proteins involved in axonal trafficking (Molteni et al., 2002, Ploughman, 2008, Erickson et al., 2012). Furthermore, physical exercise prevents other common features of aging, such as hippocampal atrophy, loss of memory and depression (Ogonovszky et al., 2005, Webster et al., 2006, Diogenes et al., 2007, Luellen et al., 2007, Lee et al., 2009, Marais et al., 2009, Erickson et al., 2012). However, the importance of physical exercise and the role of early protein aggregation in modulating axonal transport of BDNF and TrkB receptors during ageing and neurodegeneration are not well understood.

In view of this, it is hypothesized that BDNF levels, trafficking and signaling are disrupted in early neurodegeneration and physical exercise might interfere in this system.

## 2 Material and methods

Experiments were conducted in agreement with the International Guideline for Animal Experimentation care and use (Demers et al., 2006), and the Brazilian federal animal welfare law 11794/08. Procedures were approved by the research ethics committee (CEUA 121/11 and 451/11) of the Institute for Biosciences, University of Sao Paulo.

### 2.1 Animals, rotenone exposure and physical exercise

Twenty aged male Lewis rats (9 months old), supplied by the central animal facility of the Institute of Biosciences of the University of Sao Paulo, were housed in groups of 3–4 animals per conventional cage, maintained at 23 C ± 2, in an inverted 12 h light/12 h dark cycle (lights off at 6 a.m.), with free access to food and water.

Animals had osmotic minipumps (Alzet) implanted subcutaneously, between their scapulae, under anesthesia with ketamine (1.25 ml/kg) and xylazine (0.5 ml/kg). Minipumps were filled either with rotenone (Sigma, USA) dissolved in equal volumes of dimethyl sulfoxide (DMSO, Sigma, USA) and polyethylene glycol (PEG, Sigma, USA) which was delivered at the rate of either 1 mg/kg/day during 8 weeks (n = 10 animals), or only DMSO:PEG (1:1, n = 10 animals) as control. Minipumps were replaced at week 4 to guarantee 8 weeks of treatment.

One week after minipumps implant, all Lewis rats were familiarized to treadmill during 3 weeks, 3×/week, 30 min/day. After this, rats were preselected according to their ability to run in a treadmill and allocated to aerobic training (EXE, 50–60% of maximal exercise capacity, 5 days/week, 40 min/day, during 6 weeks) or kept sedentary (SED).

Maximal exercise capacity was determined by the maximal exercise test (starting at 0.3 km/h, with increments of 0.3 km/h every 3 min until exhaustion), which was repeated every 2 weeks in order to maintain training intensity.

Those rats that did not run or stopped running during protocol were excluded from the analysis. Rats were then divided in 4 groups (n = 5): DMSO-SED, DMSO-EXE, ROT-SED and ROT-EXE.

After treatment, animals were euthanized and their hippocampus removed and stored at −20 °C in protein extraction buffer (400 μl of PBS, pH 7.4, containing 1% NP40, 0.5% sodium deoxycholate, 1% SDS, 1 mM EDTA, 1 mM EGTA and 1% protease inhibitor cocktail, Sigma).

### 2.2 Primary neuronal cell culture and rotenone exposure

Cell culture method was described in detail elsewhere (Kivell et al., 2001). Briefly, for each experimental group, 20 neonatal (1 day-old) Lewis rats were decapitated and their hippocampus was dissected out, dissociated in sterile cold solution consisted of 120 mM NaCl, 5 mM KCl, 1.2 mM KH_2_PO_4_, 1.2 mM MgSO_4_, 25 mM NaHCO_3_, 13 mM glucose, pH 7.2, and subjected to mechanical and chemical cell decoupling. Solution containing cells was centrifuged at 300 g for 5 min. Supernatant was discarded and cells were suspended in Neurobasal A medium (Gibco) supplemented with 0.25 mM Glutamax (Gibco), 2% B27 (Gibco); 0.25 mM l-Glutamine (Sigma) and 40 mg/L Gentamicin (Gibco).

Cells were plated on 24-well plate (Nunc) or confocal dishes, coated with poli-_d_-lysine, at the concentration of 1800 cells/mm^2^. Cultures were kept in a humidified incubator with 5% CO_2_ at 37 °C for nine days with medium changed every three days of cultivation.

Rotenone was prepared with DMSO and diluted in culture medium applied to cell cultures in concentrations of 0.3, 0.5 and 1.0 nM for 48 h, control cultures were exposed to DMSO diluted in culture medium, all cells were exposed to at most 0.001% DMSO.

### 2.3 Western blot

Brain tissues were homogenized in extraction buffer and centrifuged at 14000 rpm for 20 min; the resulting supernatant was fractionated by SDS-PAGE (15 μg of protein/lane) using a 12% tris-HCl gel at 100 V for 1 h. Proteins were transferred to nitrocellulose membrane in transfer buffer (25 mM Tris, 190 mM glycine, 10% methanol) for 1 h at 100 V at 4 °C. Membranes were blocked for 1 h at room temperature in Tris-Buffered saline containing Tween 20 (TBS-T; 50 mM Tris, pH 8.0, 133 mM NaCl, 0,2% Tween 20) with 5% non-fat dry milk or 3% BSA (Sigma).

Blots were incubated with primary antibodies against BDNF (N-20, sc-546, Santa Cruz, 1/500); TrkB (H-181, sc-8316, Santa Cruz, 1/1000); SLP-1 (sc-136480, Santa Cruz, 1/2000); CRMP-2 (C2993, Sigma, 1/7000); Rab-27B (R-4655, Sigma, 1/1000); EDH-4 (NBP1-54873, Novus, 1/1000); KIF1B (L-20, sc-18739, Santa Cruz, 1/200); Dynein (R-325, sc-9115, Santa Cruz, 1/200); Dynactin (H-300, sc-11363, Santa Cruz, 1/400); AKT (9272, Cell Signaling, 1/2000); pAKT (ser-473, Cell Signaling, 1/750), NeuN (MAB377, Millipore, 1/500), JNK (9258, Cell Signaling, 1/1000) or p53 (2524, Cell Signaling, 1/500), in 3% non-fat dry milk or 1% BSA in TBS-T, overnight at 4 °C, followed by horseradish peroxidase-conjugated anti-mouse (1/6000, Amersham), anti-goat (1/5000, Amersham) or anti-rabbit (1/10000, Amersham). Secondary antibodies incubations were performed at room temperature during 1 h.

Development was done after 5-min incubation with enhanced chemiluminescence reagent (Millipore) and exposure to chemoluminescence sensitive films (Hyperfilm ECL, Amersham Biosciences). After development, blots were incubated with anti-beta-actin antibody (sc-47778, Santa Cruz, 1/1000) during 1 h at room temperature, followed by horseradish peroxidase conjugated anti-mouse (1/6000, Amersham), 1 h at room temperature, and were developed as previously described. Beta actin values were used for loading control and normalization.

Membranes for JNK and p53 analysis were stained with Ponceau S solution (Sigma), prior to blocking with 5% non-fat dry milk, for JNK and p53 normalization.

Films and Ponceau stained membranes were quantified using Image J software (NIH).

### 2.4 Immunocytochemistry for hyperphosphorylated Tau and MAP-2

Neurons were fixed in 50% methanol and 50% acetone for 10 min at −20 °C, rinsed with PBS and permeabilized with 0.2% triton X-100 in PBS for 30 min. Non specific binding was blocked with 2% normal goat serum and 4% BSA in PBS for 30 min and then incubated overnight with primary antibodies against hyperphosphorylated Tau (1/1000, Sigma, Ser 199/202, T6819, raised in rabbit) or MAP2 (neuron marker, 1/1000, sc 74422; Santa Cruz, raised in mouse) in 1% NGS, 2% BSA and 0.2% triton X-100. Dishes were washed with cold PBS followed by incubation with FITC or Texas red conjugated secondary antibody for 2 h. Cells were mounted with mounting medium containing DAPI (4′,6-diamidino-2-phenylindole, Vector laboratories).

### 2.5 Axonal trafficking of BDNF-containing vesicles

Primary neurons were transfected with pβ-actin-BDNF-mRFP plasmid (kindly provided by Professor Michael A. Silverman, Simon Fraser University, Canada) using Lipofectamine 2000. Movement of BDNF-containing dense core vesicles was evaluated 48 h post-transfection in neurons cultured in phenol red free medium, using a Carl Zeiss LSM780 inverted Multiphoton microscope with an integrated live cell chamber allowing 5% of CO_2_ and 37 °C environment, using the 63× objective.

Individual axons were identified according with their distinguishable morphological features, BDNF transport was evaluated in 100 μM length area through time-series of 121 images taken with 1 s interval and exposure of 100 ms from three neurons of different fields, per sample plate (n = 3 plates from 5 different cell cultures, or as indicated in graph bars). Movies were built using ImageJ and analyzed using difference tracker plugin as described by Andrews et al. (2010). Total BDNF moving tracks were accessed as well as the direction of these tracks allowing the evaluation of retrograde or anterograde trafficking.

### 2.6 Neurons morphology and neurites length analysis

Neurons morphology and neurites length were accessed through immunocytochemistry assays accordingly with the previously described protocol (Ho et al., 2011). Briefly, after exposure to rotenone neurons were fixed and permeabilized as described for immunocytochemistry for hyperphosphorylated Tau and MAP-2. Cells were incubated overnight with primary antibody against MAP2 (1/1000) followed by incubation with mouse FITC conjugated secondary antibody for 2 h, mounted with mounting medium containing DAPI and imaged on Carl Zeiss LSM780 inverted Multiphoton microscope using the 20× objective. Images from four randomly selected fields per plate from 3 different cultures were taken and the quantitative analysis of immunofluorescence data was performed using Image J (NIH) and the plug-in NeurphologyJ, as described previously, using MAP2 as a marker for neurites (Ho et al., 2011).

### 2.7 Statistical analysis

One-way or two-way ANOVA followed by Bonferroni post-hoc test accessed through GraphPad Prism (GraphPad Software Inc., version 5.00, CA) was employed to analyze results. Data are expressed as percent of control (DSMSO) ± standard deviation (SD). A p-value ≤ 0.05 was considered to indicate statistically significant differences.

## 3 Results

### 3.1 Hyperphosphorylated Tau and cell viability remains unchanged in the presence of low doses of rotenone and moderate physical activity

**Fig. 1:**
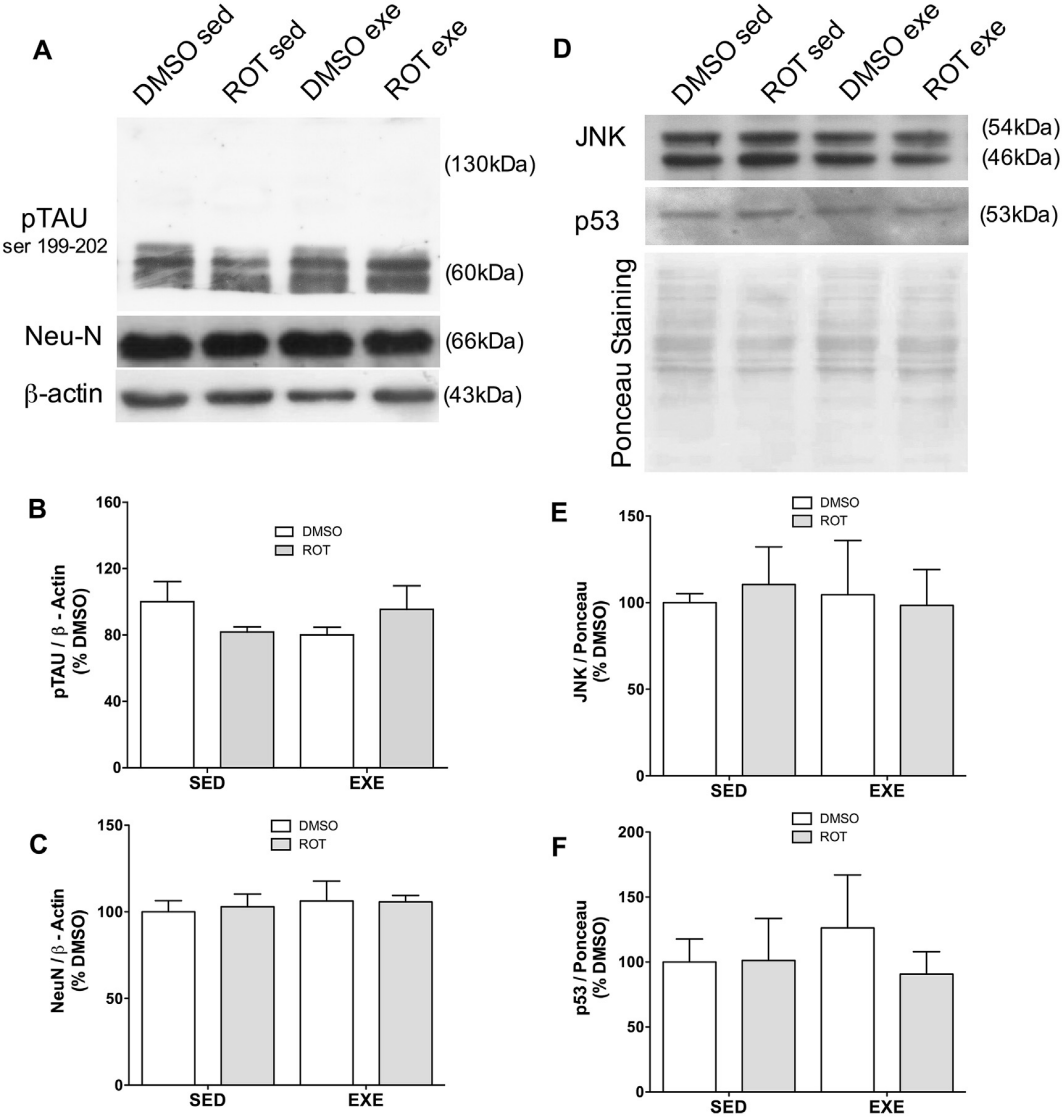
Hyperphosphorylated Tau (pTAU), Neu-N, JNK and p53 levels in hippocampus of 11 months old rats exposed to rotenone (ROT, 1 mg/kg/day) or DMSO (control), during 8 weeks, and exercised during 6 weeks (EXE) or left sedentary (SED). It is shown the absence of high molecular weight bands for pTau (A) and the lack of alterations for proteins associated with cell loss (A, C–F). Values were normalized by beta-actin (B and C) or Ponceau staining (E and F), expressed as percentage of control (DMSO sedentary) ± S.D. Means were compared through two-way ANOVA followed by Bonferroni ad-hoc test. n = 5.

These experiments were done to evaluate the effects of low doses of rotenone upon Tau aggregation and cell death, in order to ensure the evaluation of early neurodegeneration. Aged Lewis rats exposed to rotenone do not show significant differences in Tau hyperphosphorylation (Fig. 1A and B). Moderate physical exercise also does not change pTau levels (Fig. 1A and B) or the aggregation as demonstrated by the absence of higher molecular weight bands in Fig. 1A.

Neuronal loss was evaluated by quantification of Neu-N, JNK and p53 levels, it was shown that rotenone at low concentrations, as well as moderate physical activity do not change neuron viability (Fig. 1A, C–F).

### 3.2 BDNF and TrkB levels in the hippocampus of exercised aged Lewis rats during early neurodegeneration

**Fig. 2:**
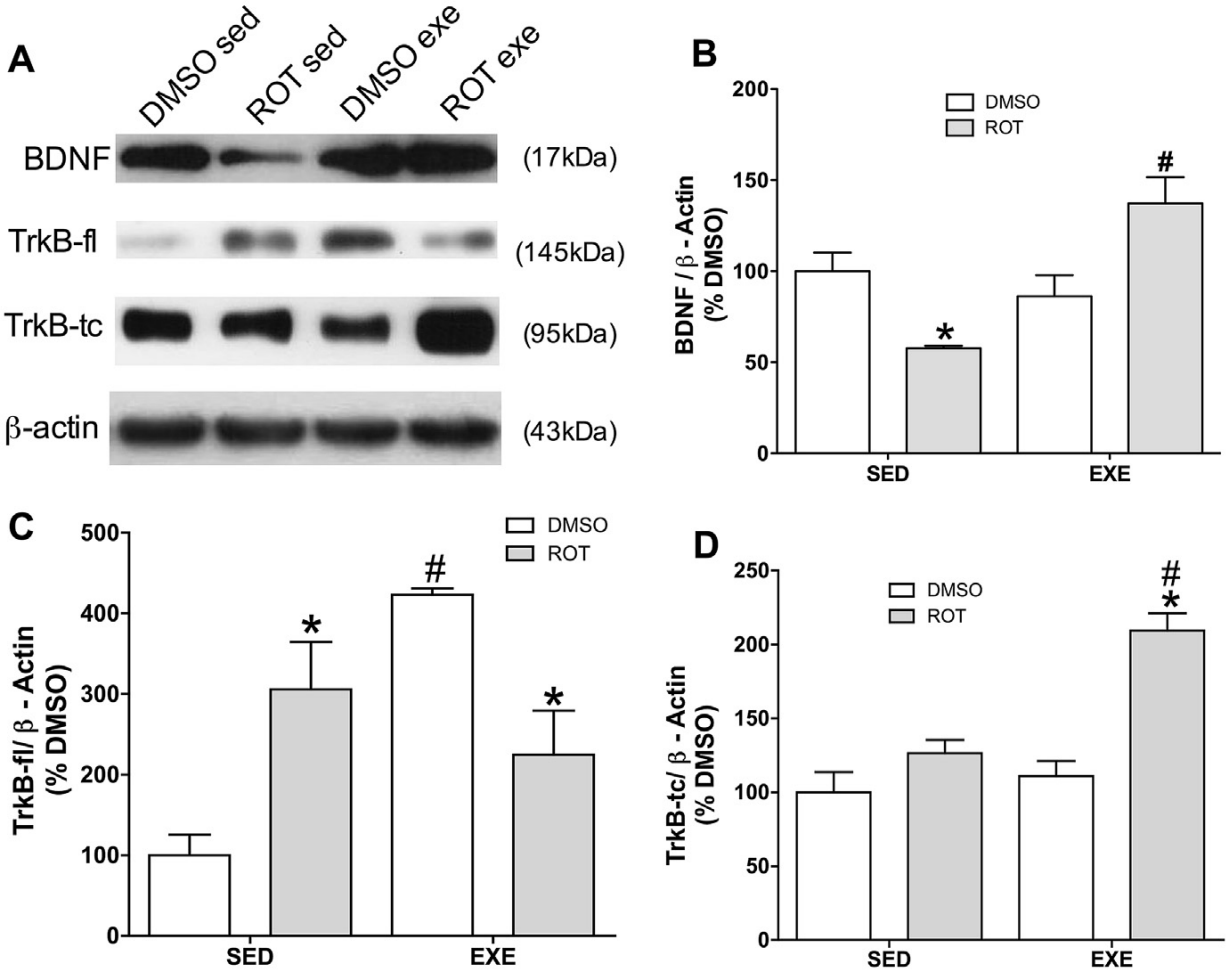
BDNF, TrkB-fl (full length) and TrkB-tc (truncated) levels in hippocampus of 11 months old rats exposed to rotenone (ROT, 1 mg/kg/day) or DMSO (control), during 8 weeks, and exercised during 6 weeks (EXE) or left sedentary (SED). Representative images of immunoblot bands corresponding to BDNF, TrkB-fl, TrkB-tc and beta-actin (A). Quantification of BDNF (B), TrkB-fl (C) and TrkB-tc (D) expression. Values were normalized by beta-actin, expressed as percentage of control (DMSO sedentary) ± S.D. Means were compared through two-way ANOVA followed by Bonferroni ad-hoc test. *p < 0.05 as compared with the DMSO from the same physical activity protocol (sedentary or exercised); #p < 0.05 as compared with sedentary exposed to the same drug protocol (DMSO or Rotenone) n = 5.

BDNF levels decrease in hippocampus of aged Lewis rats in the absence of large aggregate-like p-Tau, however physical exercise prevents this decrease and raises the levels of BDNF about 40% over DMSO sedentary rats (Fig. 2A and B).

TrkB full length increases 200% in the absence of aggregate-like p-Tau, physical activity alone also increases this TrkB isoform (300%), combination of rotenone and physical exercise promotes an increase of 150% of TrkB-fl receptor (Fig. 2A and C).

On the other hand, TrkB truncated, which might have inhibitory effects upon TrkB-fl, was also elevated 150% in hippocampus of aged Lewis rats exposed to rotenone and to physical activity practice (Fig. 2A and D).

### 3.3 Trafficking of TrkB vesicles in the hippocampus of exercised aged Lewis rats during early neurodegeneration

**Fig. 3:**
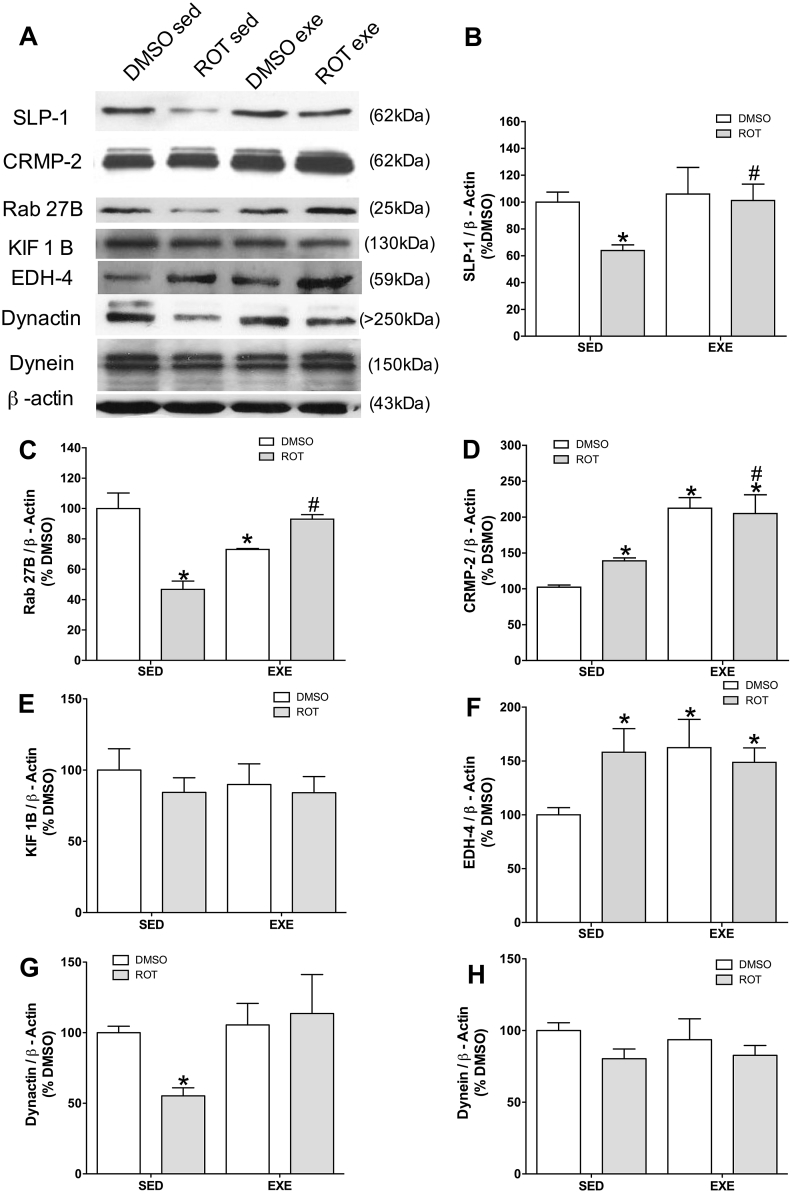
Trafficking proteins levels in hippocampus of 11 months old rats exposed to rotenone (ROT, 1 mg/kg/day) or DMSO (control), during 8 weeks, and exercised during 6 weeks (EXE) or left sedentary (SED). Representative images of immunoblot bands corresponding to SLP-1, Rab27B, CRPM-2, KIF1B, EDH-4, Dynactin, Dynein and beta-actin (A). Quantification of SLP-1(B), Rab27B (C), CRMP-2 (D), KIF1B (E), EDH-4 (F), Dynactin (G) and Dynein (H) expression. Values were normalized by beta-actin, expressed as percentage of control (DMSO sedentary) ± S.D. Means were compared through two-way ANOVA followed by Bonferroni ad-hoc test. *p < 0.05 as compared with the DMSO from the same physical activity protocol (sedentary or exercised); #p < 0.05 as compared with sedentary exposed to the same drug protocol (DMSO or Rotenone) n = 5.

The levels of SLP-1, CRMP-2, Rab27bB, KIF1B were measured in order to evaluate anterograde trafficking of TrkB receptors. The levels of specific protein complex related to anterograde trafficking of TrkB vesicles (SLP-1 and Rab27B) decreased before major hyperphosphorylation of Tau (35% for SLP-1 and 50% for Rab27B), whereas physical activity prevented this decrease (Fig. 3A, B and C). CRMP-2 increased 40% in early neurodegeneration, which was potentialized (raise of 100%) by physical activity (Fig. 3A and D). KIF1B did not change in the present experimental protocol (Fig. 3A and E).

Retrograde trafficking of TrkB receptors was evaluated by the expression of EDH-4, dynactin and dynein. EDH-4 is 80% increased in early neurodegeneration and in the presence of physical activity (Fig. 3A and F). Dynactin decreased 45% in early neurodegeneration (Fig. 3A and G) and dynein was not altered in the present study (Fig. 3A and H).

### 3.4 Immunolabeling of hyperphosphorylated Tau in cultured hippocampal cells

**Fig. 4:**
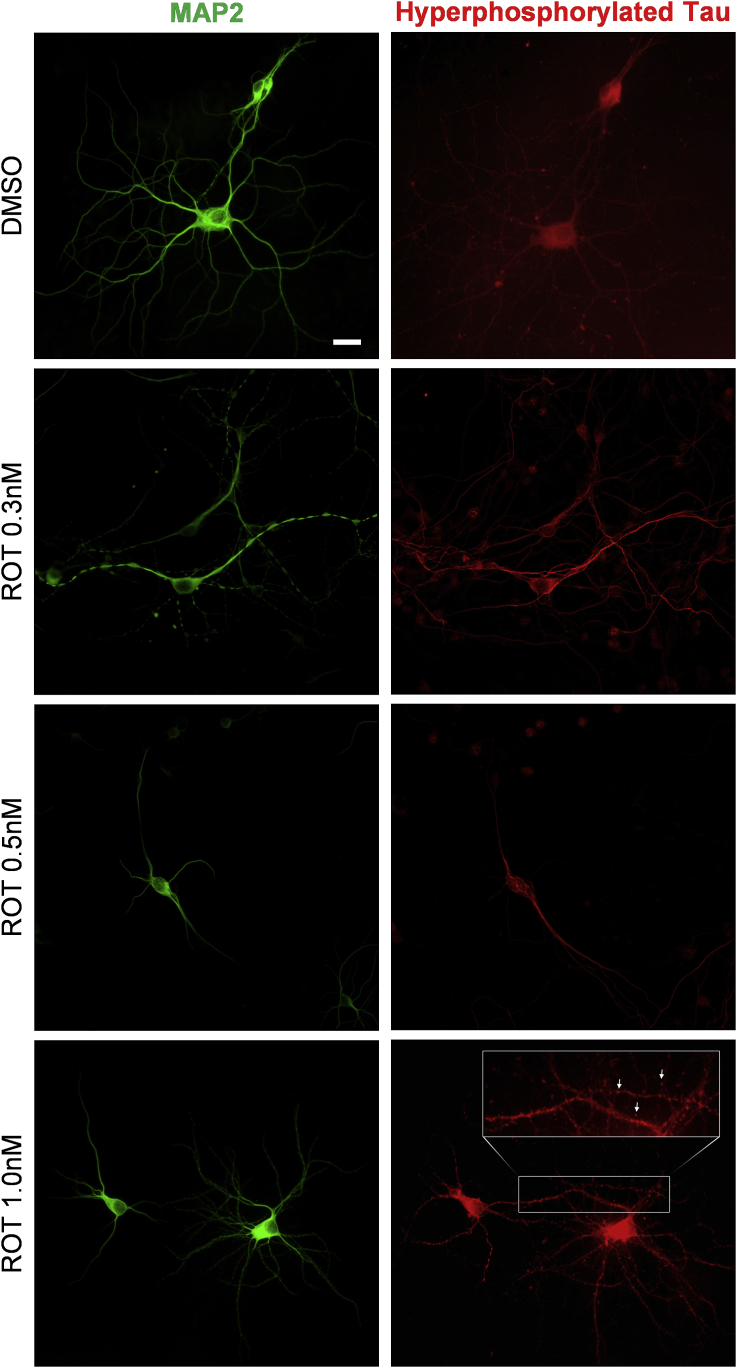
Illustrative digital images of cultured hippocampal neurons labeled with antibodies against MAP2 and hyperphosphorylated Tau (p-TAU) after exposure to DMSO (control), 0.3, 0.5 or 1 nM of rotenone during 48 h. Note the decrease in size and number of neuronal branches evidenced after 1 nM rotenone treatment. Dense p-Tau aggregates-like are found in neuronal cells exposed to 1 nM rotenone (arrows). Scale bar = 50 μm.

Immunocytochemical labeling of hippocampal cultures showed that cultures exposed to 1 nM rotenone present p-Tau labeling mainly localized towards the cell body, suggesting mislocation (Fig. 4). Rotenone at 1 nM also promoted aggregate-like pattern of p-Tau labeling, demonstrated by the puncta labeling (Fig. 4, arrows).

### 3.5 Decrease in trafficking of BDNF-containing vesicles in early neurodegeneration and during cell death in primary hippocampal cell culture

**Fig. 5:**
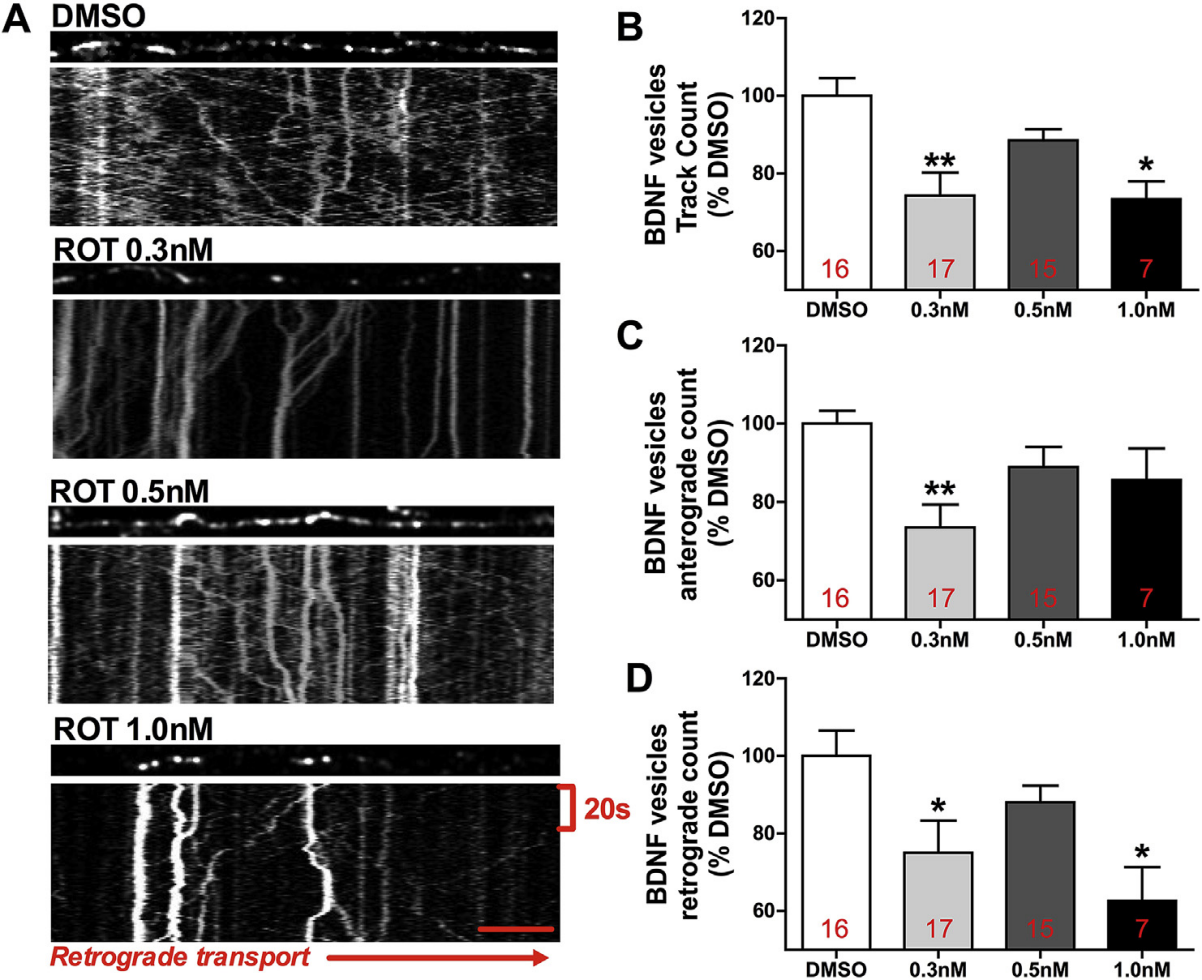
Axonal trafficking of BDNF-containing vesicles in hippocampal primary cell cultures exposed to rotenone (ROT, 0.3 nM, 0.5 nM or 1.0 nM) or DMSO (control), during 48 h. Representative kymographs comparing axonal trafficking of BDNF-containing vesicles in DMSO, 0.3 nM ROT, 0.5 nM ROT or 1.0 nM ROT (A). Quantification of BDNF vesicles track count (B), BDNF vesicles anterograde count (C) and BDNF vesicles retrograde count (D). Values were expressed as percentage of control (DMSO) ± S.D. Means were compared through one-way ANOVA followed by Bonferroni ad-hoc test. *p < 0.05; **p < 0.01 as compared with DMSO. Number in each column represents the number of cells considered to count vesicles movements.

After observing alterations in the levels of proteins associated with trafficking of TrkB vesicles, the next step was to analyze the transport of BDNF-containing vesicles in live cells. Axonal trafficking of BDNF-containing vesicles was decreased (30%) in the absence of major hyperphosphorylation of Tau (ROT 0.3 nM, Fig. 5A and B). It is interesting to note that during the increase of p-Tau (0.5 nM rotenone) there is normalization of axonal trafficking (Fig. 5A and B). In the presence of large aggregate-like conformation of p-Tau and beginning of cell death (ROT 1.0 nM) trafficking of BDNF vesicles is impaired (Fig. 5A and B). Anterograde and retrograde trafficking is decreased during early neurodegeneration (Fig. 5A, C and D), whereas this dysfunction at late stages of p-Tau accumulation is present only in retrograde trafficking (Fig. 5A and D).

### 3.6 BDNF and TrkB receptors levels in primary hippocampal cell culture during accumulation of p-Tau

**Fig. 6:**
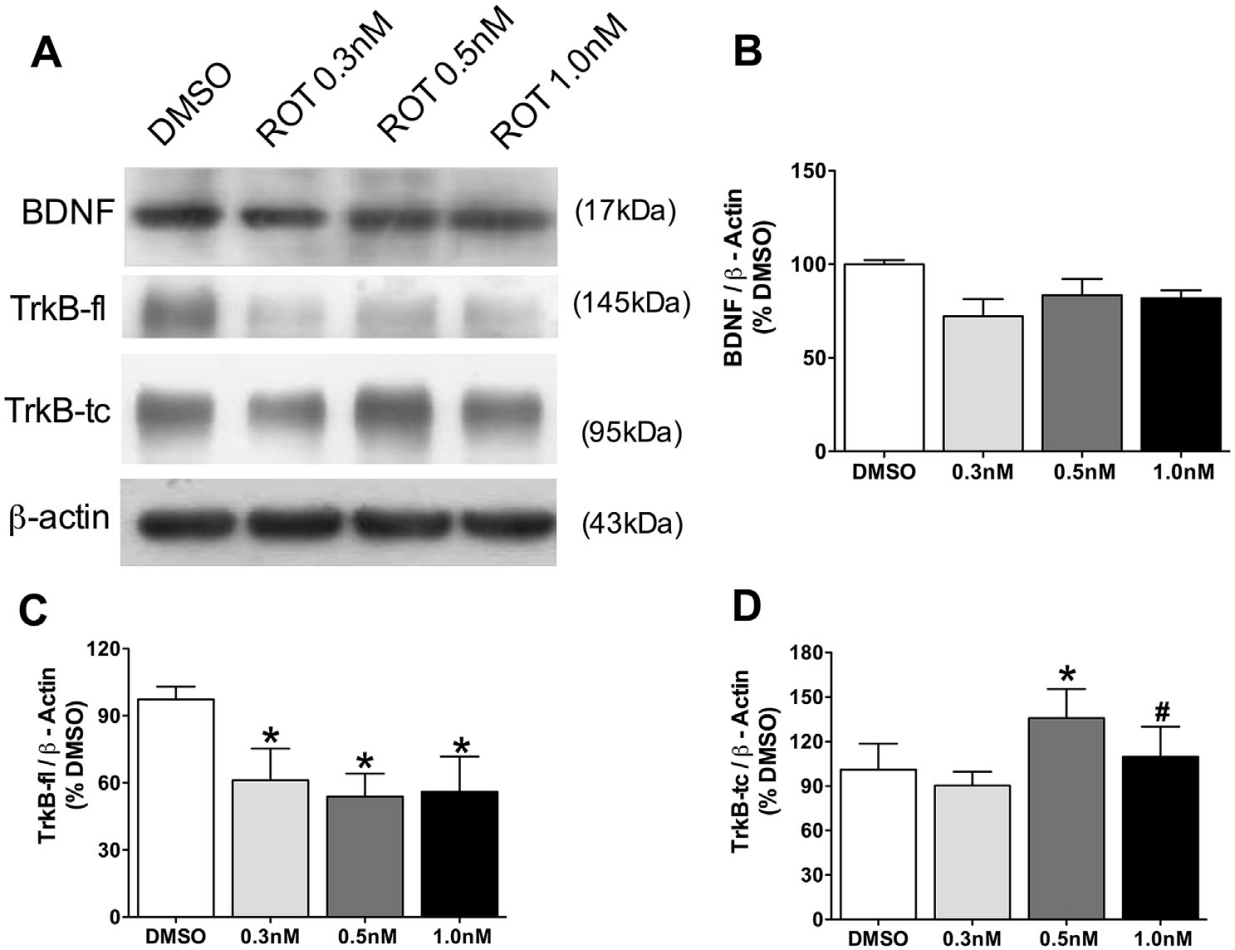
BDNF, TrkB-fl and TrkB-tc levels in hippocampal primary cell cultures exposed to rotenone (ROT, 0.3 nM, 0.5 nM or 1.0 nM) or DMSO (control), during 48 h. Representative images of immunoblot bands corresponding to BDNF, TrkB-fl, TrkB-tc and beta-actin (A). Quantification of BDNF (B), TrkB-fl (C) and TrkB-tc (D) expression. Values were normalized by beta-actin, expressed as percentage of control (DMSO) ± S.D. Means were compared through one-way ANOVA followed by Bonferroni ad-hoc test. *p < 0.05 as compared with DMSO; #p < 0.05 as compared with 0.3 nM n = 3 independent cultures, run in technical triplicates.

In order to compare the effects of rotenone upon the levels of BDNF and TrkB receptors between aged rats and cell culture, these parameters were evaluated by western blot. BDNF levels did not change after rotenone exposure, although at 0.3 nM (before to accumulation of p-Tau) there is a tendency of decrease (30%) in its levels (p = 0.07, Fig. 6A and B). TrkB full length decreased 40% before and during to accumulation of p-Tau (Fig. 6A and C), whereas truncated isoform of TrkB receptor increases in the presence of aggregate-like p-Tau (0.5 and 1 nM rotenone, Fig. 6A and D).

### 3.7 Levels of proteins involved in anterograde trafficking of TrkB in cultured hippocampal cells

**Fig. 7:**
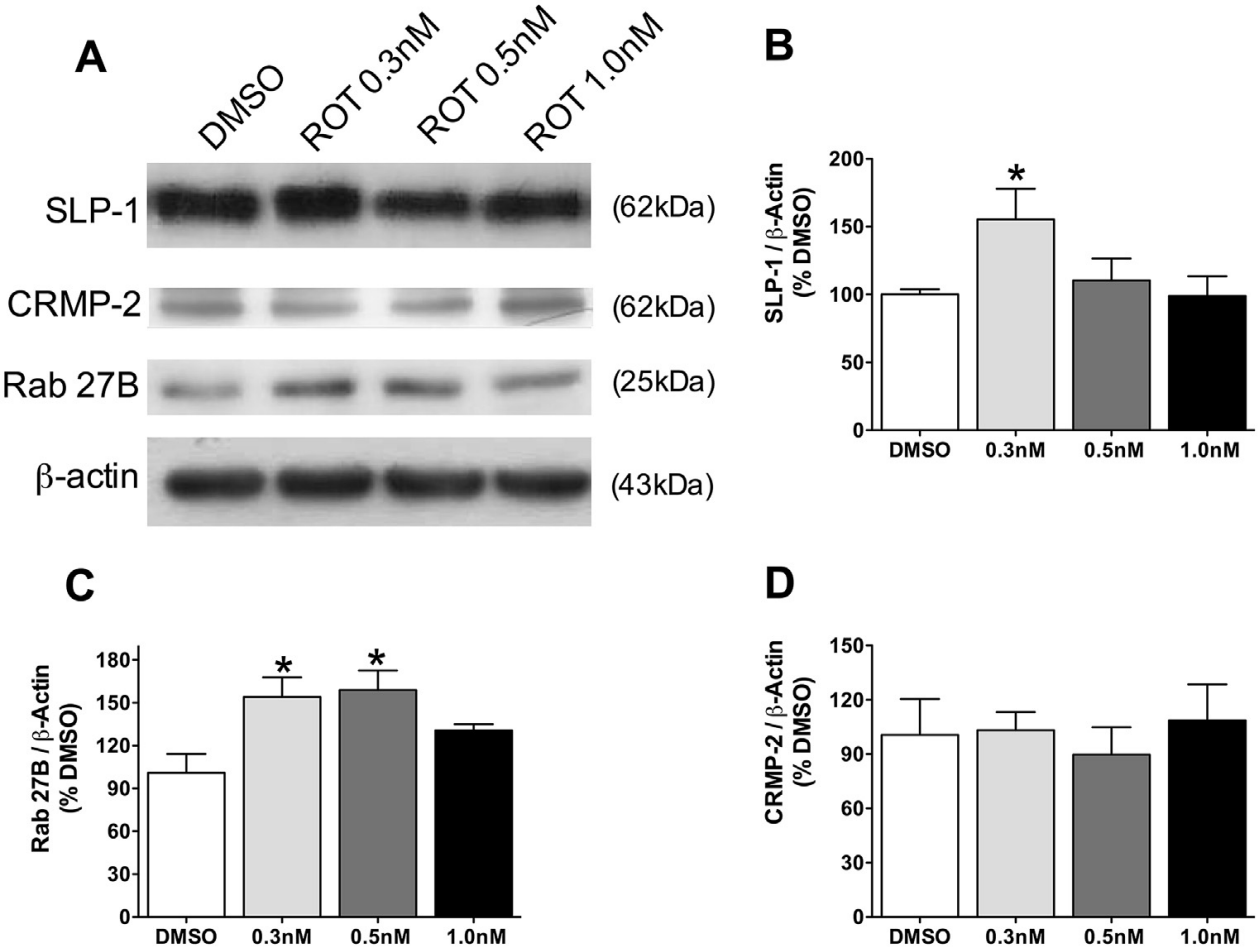
Molecular proteins levels in hippocampal primary cell cultures exposed to rotenone (ROT, 0.3 nM, 0.5 nM or 1.0 nM) or DMSO (control), during 48 h. Representative images of immunoblot bands corresponding to SLP-1, Rab27B, CRPM-2, and beta-actin (A). Quantification of SLP-1(B), Rab27B (C) and CRMP-2 expression. Values were normalized by beta-actin, expressed as percentage of control (DMSO) ± S.D. Means were compared through one-way ANOVA followed by Bonferroni ad-hoc test. *p < 0.05 as compared with DMSO. n = 3 independent cultures, run in technical triplicates.

Trafficking of BDNF receptors was also evaluated by studying the levels of proteins involved in this transport. Complex SLP-1/Rab27B is increased before accumulation of p-Tau and during the early aggregation (ROT 0.3 nM and 0.5 nM, respectively, Fig. 7A, B and C), whereas there is no change in the levels of these proteins after p-Tau deposits (ROT 1.0 nM, Fig. 7A–C). CRMP-2 levels did not change after rotenone exposure (Fig. 7A and D).

### 3.8 TrkB-fl/BDNF signaling pathway

**Fig. 8:**
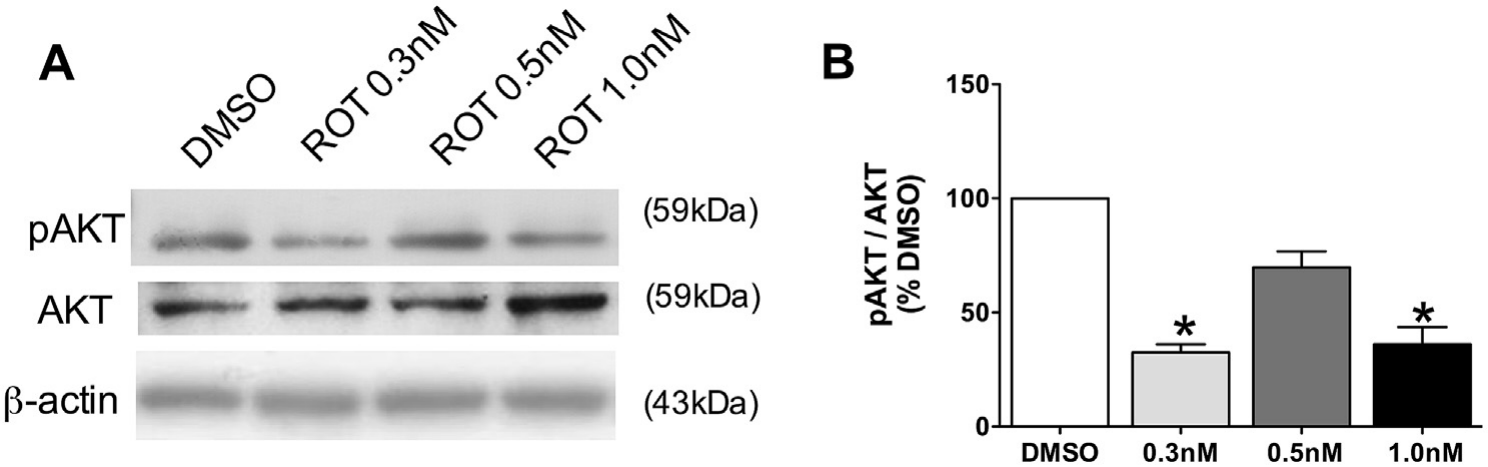
Phosphorylated AKT and AKT levels in hippocampal primary cell culture exposed to rotenone (ROT, 0.3 nM, 0.5 nM or 1.0 nM) or DMSO (control), during 48 h. Representative images of immunoblot bands corresponding to pAKT, AKT and beta-actin (A). Quantification of pAKT/AKT ratio (B) expression. Values were normalized by beta-actin, expressed as percentage of control (DMSO) ± S.D. Means were compared through ane-way ANOVA followed by Bonferroni ad-hoc test. *p < 0.05 as compared with DMSO. n = 3 independent cultures, run in technical triplicates.

Although changes in TrkB-fl and BDNF levels were found in the present study, the real physiological significance is measured by activation of intracellular cascades. TrkB-fl/BDNF signaling pathway was investigated by measuring phosphorylation of AKT. It was demonstrated a decrease in pAKT levels during rotenone exposure, with the lowest levels (decrease of 70%) being encountered in the absence of p-Tau deposits (0.3 nM rotenone, Fig. 8A and B).

### 3.9 Neurite quantification

**Fig. 9:**
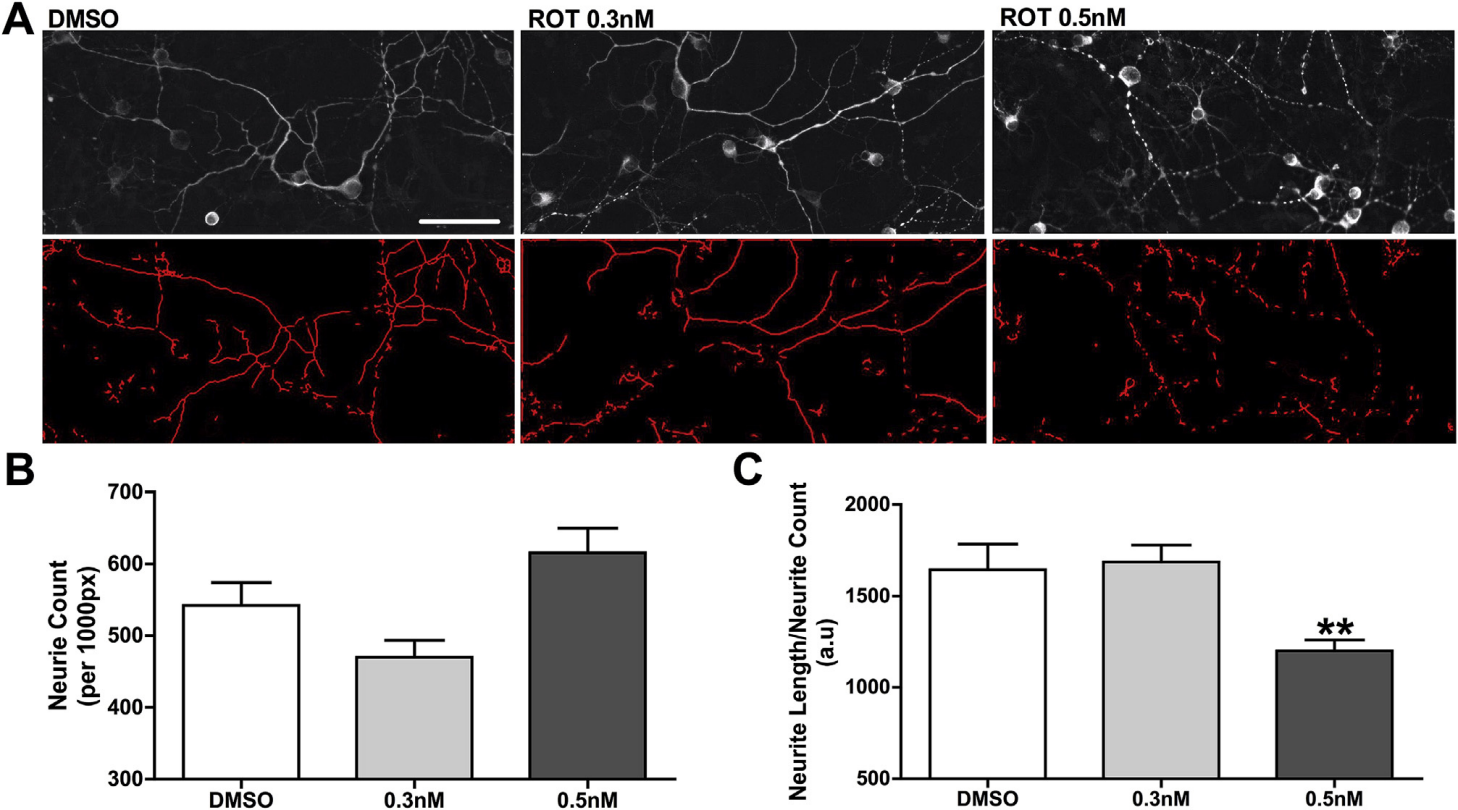
Neurons morphology and neurites length. Illustrative digital images of hippocampal primary cell culture exposed to rotenone (ROT, 0.3 nM or 0.5 nM) or DMSO (control), during 48 h (A), labeled with anti MAP-2 (upper panels) and subjected to ImageJ for neurite track (bottom panels). Quantification of neurite count (B) and neurite length (C). Means were compared through one-way ANOVA followed by Bonferroni ad-hoc test. **p < 0.01 as compared with DMSO. n = 3 independent cultures, run in technical triplicates. Bar scale = 300 μm.

The latter consequence of disturbance in BDNF system may be fragmentation of neuron prolongations. To test this hypothesis, neurite count and length were quantified. Confocal microscopy of primary hippocampal cell culture showed that accumulation of insoluble p-Tau triggered expressive neurite fragmentation compared to control (Fig. 9A). Quantification of branch number demonstrated an increase of 10% in neurite count after increased p-Tau (ROT 0.5 nM, Fig. 9B), accompanied by decrease in neurite length (Fig. 9C), suggesting that dendrite/axon fragmentation occurs during p-Tau accumulation promoted by rotenone exposure.

## 4 Discussion

In the present study it was demonstrated, for the first time, that alterations in BDNF/TrkB levels, signaling and trafficking precede any change in hyperphosphorylation of Tau in hippocampus of aged rats exposed to rotenone. It was also shown that moderate physical activity prevents the impairment of BDNF system in aged rats, which may be one contributing factor to delay neurodegeneration (Colcombe et al., 2003).

Rotenone is a specific inhibitor of mitochondrial complex I, it was employed in the present study to simulate the different phases of protein aggregation, as can be found in early neurodegeneration. It was demonstrated previously that rotenone is able to promote “*in vitro”* and “*in vivo”* aggregation and hyperphosphorylation of tau without causing cell death (Hoglinger et al., 2005, Radad et al., 2008, Ullrich and Humpel, 2009, Chaves et al., 2010, Chaves et al., 2013, Melo et al., 2013). Several recently published studies confirmed the accuracy of Neu-N levels, measured by Western blot, alone or compared with immunolabeled neurons or caspase 3 levels, to infer about cell loss (Zhang et al., 2013, Murphy et al., 2014, Cheng et al., 2015, Wang et al., 2015). We went further and also quantified the levels of JNK and p53 to demonstrate that there is no neuron loss in the present experimental protocol.

Aged rats exposed to 1 mg/kg/day of rotenone, as well as physical exercise, do not present differences in p-Tau as demonstrated herein, validating the hypothesis that the changes in BDNF system occur in advance to accumulation of p-Tau. Also, in cultured hippocampal cells, exposure to 1 nM of rotenone during 48 h induces hyperphosphorylation of tau mimicking cellular dysfunctions present in AD. On the other hand, exposure to 0.3 nM rotenone does not change hyperphosphorylation of Tau, and 0.5 nM during 48 h induces oligomerization of hyperphosphorylated tau without triggering major protein aggregation (Chaves et al., 2010). Thus, rotenone exposure protocol employed in the present study for rats is comparable to the lowest concentration applied in hippocampal cell culture (0.3 nM) considering its effects upon hyperphosphorylation of Tau.

There is a number of different physical exercise protocols applied to different animal models of neurodegeneration. Results largely disagree about the effects of physical activity upon the central nervous system during the neurodegenerative process (Lin et al., 2015, Elahi et al., 2016), however the majority of these previous studies evaluated physical activity as a method to treat neurodegeneration already installed. Our present study intends to add some knowledge upon the process before the first signs of neurodegeneration appear.

BDNF signaling seems to be elevated by physical activity in the hippocampus of aged rats in the presence of a neurodegenerative insult, as previously described elsewhere (Coelho et al., 2014), the present study is of relevance since it shed light on the mechanism by which moderate physical activity may be acting to delay neurodegeneration associated to protein aggregates.

The increase in TrkB-fl receptor levels in sedentary rats during early neurodegeneration may be a response to the decreased levels of BDNF before aggregate-like p-Tau is formed, as a mechanism of feedback between these proteins. In fact, autocrine mechanism of BDNF signaling has long been described (Kokaia et al., 1993), in that context we hypothesize that there might be a compensatory conversation between BDNF and its receptors in the animal model. This is evidenced by the decrease of TrkB levels after physical exercise that increased BDNF levels in the presence of rotenone. Moreover, physical activity improves BDNF signaling by elevating BDNF and TrkB-fl levels during early neurodegeneration.

Specifically about the function of truncated isoform of TrkB, it is still controversial in literature. TrkB-tc may be associated with recycling of BDNF (Fenner et al., 2014), other studies demonstrate that TrkB-tc decrease the function of TrkB-fl (Salehi et al., 2006). It seems that the role of TrkB-tc depends upon the presence of BDNF, since in the presence of this neurotrophin, TrkB-tc is associated with cell survival response, whereas in the absence of BDNF TrkB-tc may contribute to cell death and acts as dominant negative inhibitor of TrkB-fl (Gomes et al., 2012).

In the present study the increase of TrkB-tc was accompanied by decrease of TrkB-fl, without changes in BDNF levels, which was related to neurite fragmentation, corroborating the idea of an antagonistic relation between the full length and truncated isoform of TrkB.

In agreement with the idea of BDNF signaling improvement by physical activity during early neurodegeneration, in the present study it was demonstrated for the first time that physical exercise prevents the decrease in trafficking proteins associated with BDNF and TrkB receptors anterograde and retrograde axonal transport, present during neurodegeneration (Chaves et al., 2013, Melo et al., 2013), these previously published data refers to aged rats exposed to one month of rotenone exposure, the present study shows data from aged rats exposed to two months of rotenone, this may explain the supposed difference in the expression of KIF1B and dynactin.

Primary hippocampal cell cultures were exposed to low and moderate doses of rotenone in order to investigate the mechanisms underlying BDNF and TrkB receptors levels and trafficking before and during hyperphosphorylation of Tau. However, since it is not possible yet to reproduce ageing in primary cell culture, and many of the results are related to ageing itself in brain of aged rats, there are several results that do not corroborate with *in vivo* data. Another important fact that must be taken into account is that rotenone delivered subcutaneously might not be as concentrated in brain as it is in plasma, furthermore, time of exposure also may not be comparable between the two experimental models.

Moreover, Perovic et al. (2013) described that hippocampal BDNF mRNA expression was lower in 12-month-old rats as compared with young rats, moreover, the levels of p-Akt and protein kinase C were decreased in aged rats. This is evidence that aging, *per se*, is an insult to BDNF system, indicating that the basal situation (control groups) cannot be compared between cultures and *in vivo* models. BDNF system in cultured neurons is affected only by rotenone, thus it seems that rotenone acts preferably upon trafficking (probably because of the presence of oligomers) and levels of TrkB receptors. Aged rats, on the contrary, are subjected to rotenone besides the aging process, in this context BDNF levels decreased (probably because there are two effectors). In summary, low levels of rotenone is able to interfere with trafficking, whereas in the presence of the aging process there are more pronounced effects upon BDNF availability. The differences encountered for cultures and aged rats is not at all invalidated, since it is demonstrated *in vitro* and *in vivo* that alteration in cell physiology is already established in the absence of Tau hyperphosphorylation.

The present study demonstrates for the first time a decrease in trafficking of BDNF-containing vesicles, mainly in retrograde direction, even in the absence of protein aggregates. Impairment of BDNF signaling is corroborated also by the decrease on TrkB-fl levels, before large p-Tau aggregate-like forms.

It is interesting to point out that the decrease in BDNF trafficking is accompanied increase of specific proteins, such as SLP-1 and Rab27B, related to trafficking of TrkB receptors. This controversy may be partially explained by the compensatory cellular feedback in response to decrease in BDNF signaling. SLP-1 and Rab27 are proteins related to anterograde trafficking of TrkB receptors, especially at the final portion of axon terminal, since these proteins use actin cytoskeleton (Johnson et al., 2012). One could hypothesize that this is an attempting to increase TrkB receptors at synaptic terminal to improve BDNF signaling.

Moreover, increased intracellular calcium concentrations are reported in the presence of Aβ oligomers (Decker et al., 2010, Gan and Silverman, 2015), which is associated with early pathological manifestations in AD (Berridge, 2011).

BDNF signaling was evaluated by the ratio of phosphorylated AKT by total AKT, confirming the decrease of BDNF signaling in the absence of aggregate-like hyperphosphorylated Tau. Reduction on pAKT/AKT ratio correlates with neuron loss in cultured primary mouse neurons, in old 3xTg-AD mice (Ghosh and Brewer, 2014) and in APP/PS1 mice (Li and Liu, 2010). Alterations in pAKT/AKT ratio have also been reported in AD (Liu et al., 2013, Ong et al., 2014). This may be of relevance for cell death, as one of the features of neurodegeneration is fragmentation of neurites, as demonstrated in the present study when the first aggregates appear (0.5 nM rotenone). BDNF signaling also involves the activation of ERK and CaMK, besides AKT, which were not evaluated in the present study; and is probably involved in the BDNF signaling after 0.5 nM rotenone exposure, as it was not observed any change in AKT whereas TrkB-fl levels were reduced at this concentration.

Is it hypothesized that biochemical changes occur before neurite fragmentation, and those imbalance would lead to fragmentation in a latter period. It was previously shown that 0.3 nM applied for 72 h has the same effects as 0.5 nM during 48 h, so the effects of 0.3nM/48 h would accumulate and cause neurite fragmentation at 72 h incubation (as demonstrated with 0.5nM/48 h). This is emphasized that BDNF trafficking was evaluated in intact cell branches, demonstrating that trafficking is not altered in the presence of the first aggregates in intact cells.

**Fig. 10:**
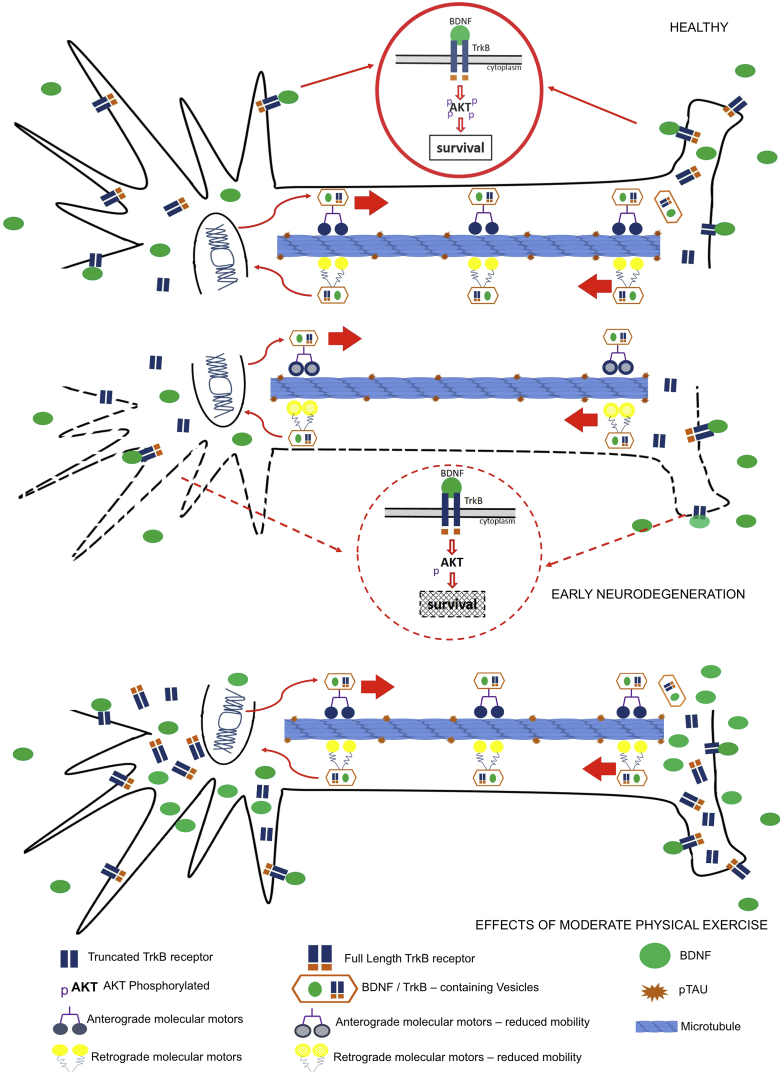
Graphical abstract depicting decrease in BDNF/TrKB signaling during early neurodegeneration, which is restored with moderate physical exercise.

In conclusion, there is evidence that BDNF signaling is impaired in the absence of large aggregate-like p-Tau forms, probably by the decrease of BDNF and TrkB vesicles trafficking, and moderate physical activity applied during early neurodegeneration may be of relevance to prevent this decline of BDNF system. Fig. 10 summarizes the results of BDNF trafficking and signaling in early neurodegeneration, as well as the effects of moderate physical activity upon BDNF pathways.
